# Inflammation: Bridging Age, Menopause and APOEε4 Genotype to Alzheimer’s Disease

**DOI:** 10.3389/fnagi.2018.00312

**Published:** 2018-10-09

**Authors:** Aarti Mishra, Roberta D. Brinton

**Affiliations:** ^1^Titus Family Department of Clinical Pharmacy, School of Pharmacy, University of Southern California, Los Angeles, CA, United States; ^2^Center for Innovation in Brain Science, University of Arizona, Tucson, AZ, United States; ^3^Department of Pharmacology, College of Medicine, University of Arizona, Tucson, AZ, United States; ^4^Department of Neurology, College of Medicine, University of Arizona, Tucson, AZ, United States

**Keywords:** inflammation, Alzheimer’s disease, menopause, APOEε4, aging

## Abstract

Neuro-inflammatory processes that contribute to development of Alzheimer’s are evident early in the latent prodromal phase and worsen during the course of the disease. Despite substantial mechanistic and clinical evidence of inflammation, therapeutic approaches targeting inflammation have failed to alter the course of the disease. Disparate results from epidemiological and clinical trials targeting inflammation, highlight the complexity of the inflammatory process. Herein we review the dynamics of the inflammatory process across aging, midlife endocrine transitions, and the APOEε4 genotype and their contribution to progression of Alzheimer’s disease (AD). We discuss the chronic inflammatory processes that are activated during midlife chronological and endocrine aging, which ultimately limit the clearance capacity of microglia and lead to immune senescence. Aging, menopause, and APOEε4 combine the three hits of a compromised bioenergetic system of menopause with the chronic low grade innate inflammation of aging with the APOEε4 dyslipidemia and adaptive immune response. The inflammatory immune response is the unifying factor that bridges across each of the risk factors for AD. Immune system regulators that are specific to stage of disease and inflammatory phenotype would provide a therapeutic strategy to disconnect the bridge that drives disease. Outcomes of this analysis provide plausible mechanisms underlying failed clinical trials of anti-inflammatory agents in Alzheimer’s patients. Further, they highlight the need for stratifying AD clinical trial cohorts based on inflammatory phenotype. Combination therapies that include targeted use of anti-inflammatory agent’s specific to the immune phenotype are considered.

## Introduction

Alzheimer’s disease (AD) is characterized by an extended prodromal phase of typically 10–20 years duration prior to clinical manifestation of cognitive decline (Amieva et al., 2008). The prodromal phase of AD consists of both- pre-stage symptoms and mild cognitive impairment (MCI) (Wilson et al., 2011). Clinical studies have shown that the prodromal phase is characterized by metabolic dysfunction, amyloid-β (Aβ) deposition in the brain, mild to moderate cognitive dysfunction, and chronic low-grade inflammation (Habeck et al., 2012; Olsson et al., 2013; Brinkmalm et al., 2014; Wirz et al., 2014; Rajan et al., 2015; Mosconi et al., 2017a,b). These hallmark pathologies have aided in the development of biomarkers predictive of disease pathogenesis. In some populations, Aβ42 is one of the first biomarkers to appear in the cerebrospinal fluid (CSF) (Jack et al., 2013; Brinkmalm et al., 2014; Calsolaro and Edison, 2016). Despite the recent and widespread recognition of neuroinflammation in the pathogenesis of AD, there has been sparse expansion of inflammation-based biomarkers and preventive strategies. Understanding the dynamic interplay between inflammation and the risk factors such as age, APOE genotype, and endocrine transition states, can aid in this process. This review addresses the interaction between inflammation and risk factors key to the pathogenesis of AD. Strategies to specifically target these processes are also considered.

## Early Activation of Inflammation and Risk for Alzheimer’S Disease

Substantial evidence documents reactive microgliosis around plaque deposition and is now a hallmark of AD pathology (McGeer et al., 1988; Mattiace et al., 1990; Xiang et al., 2006). Reactive microgliosis and neuroinflammation in AD patients is considered a consequence of Aβ plaque deposition (McGeer et al., 1987). Microgliosis in AD is evidenced both microscopically and biochemically with increased levels of the proinflammatory cytokines including tumor necrosis factor-α (TNFα), IL-6, and IL-1β (Itagaki et al., 1989; Dickson et al., 1993; Ferretti and Cuello, 2011; Eikelenboom et al., 2012; Latta et al., 2014). While the inflammatory response to Aβ plaque deposition is irrefutable, it is a late stage response in the inflammatory cascade. Indicators of earlier inflammatory responses are apparent in multiple conditions that are risk factors for later development of AD.

Associations between the occurrence of systemic infections and chronic inflammatory conditions with Alzheimer’s disease, suggests an active participation of inflammation in early stages of disease development. Patients with higher erythrocyte sedimentation rate (ESR), which is a clinical indicator of non-specific inflammation, are at greater risk of developing AD (Li et al., 2012). This is further corroborated by epidemiological studies that show that patients who suffer from chronic periodontal infection (Van Den Heuvel et al., 2007) and HIV have a higher risk of developing AD (Stanley et al., 1994; Alisky, 2007; Xu and Ikezu, 2009; Chakradhar, 2018). Recapitulating the clinical effect, Krstic et al. (2012) established an animal model that displayed AD like neuropathology by inducing chronic inflammation prenatally using a viral antigen, thereby showing that chronic inflammation potentiates the development of AD. Traumatic brain injury (TBI) also increases the risk of developing AD. Lesions that develop during TBI lead to an acute inflammatory response that includes microglial activation to facilitate debris removal and neuroprotection (Van Den Heuvel et al., 2007; Breunig et al., 2013; Habib et al., 2014). Incomplete resolution of the acute inflammatory response in TBI, however, is often followed by hypoxia and oxidative stress, which leads to the chronic activation of microglia and the release of neurotoxic proinflammatory cytokines (Van Den Heuvel et al., 2007; Breunig et al., 2013; Habib et al., 2014).

Chronic inflammatory conditions such as autoimmune disorders alter the risk of development of dementia. A recent study found patients admitted to the hospital for an autoimmune disorder have greater risk for subsequent hospitalization due to dementia (Wotton and Goldacre, 2017). This association was particularly significant for multiple sclerosis and systemic lupus erythematosus for AD. While patients with rheumatoid arthritis (RA) had a reduced risk of developing Alzheimer’s disease, they had an increased risk of vascular dementia (Wotton and Goldacre, 2017). Multiple studies indicate that AD incidence is lower in persons with RA (Policicchio et al., 2017). Some attribute this reduction in incidence to the regular use of non-steroidal anti-inflammatory drugs (NSAIDs) (McGeer et al., 1996; Etminan et al., 2003). An alternative mechanism involves upregulation of granulocyte macrophage-colony stimulating factor (GM-CSF) with a probable gain of function in myeloid cells, thus enabling effective debris clearance is also hypothesized to reduce the incidence of AD in RA patients (McGeer et al., 1996; Boyd et al., 2010). In mice, increased levels of GM-CSF (both intrahippocampal and subcutaneous administration) significantly reduced amyloidosis and reversed cognitive impairment (McGeer et al., 1996; Boyd et al., 2010). More recent findings indicate that RA and risk of AD can be stratified based on treatment. Case-controlled study conducted on electronic medical records from 8.5 million commercially insured adults, indicate that RA patients treated with an anti-TNFα therapy, etanercept, had a lower risk of AD whereas those on other anti-inflammatory agents had increased risk of AD (Chou et al., 2016).

Disparate results from epidemiological studies and randomized clinical trials highlight the complexity of response to anti-inflammatory agents (Thal et al., 2005). Epidemiological analyses indicated that long-term NSAIDs users have a lower risk of developing AD (McGeer et al., 1996; Vlad et al., 2008). Based on epidemiological findings, a clinical study – ADAPT (Alzheimer’s Disease Anti-Inflammatory Prevention Trial) was conducted in cognitively intact elderly individuals with a family history of AD. In this trial, the selective cyclooxygenase-2 (COX-2) inhibitor Celecoxib and non-selective COX inhibitor Naproxen, were used as preventive therapies. The trial was discontinued 15 months after randomization, due to the increased cardiovascular risk of these therapies. On extended follow-up after 7 years, treatment with celecoxib or naproxen for 1–3 years did not prevent cognitive decline (Group, 2007, 2008; Breitner et al., 2011; Alzheimer’s Disease Anti-inflammatory Prevention Trial Research Group, 2013). Collectively, these findings indicate that timing of NSAID treatment for chronic inflammatory conditions are a critical factor impacting the efficacy of NSAID therapy to prevent or delay progression of Alzheimer’s disease (McGeer et al., 1996; Vlad et al., 2008).

Discrepancies between the epidemiological and clinical trial findings indicate the need for greater refinement in considering patient populations and anti-inflammatory therapies. Elucidating the inflammatory phenotype which emerges during the progression of AD requires consideration of the triggers that initiate chronic inflammation. In the sections below, we address how age, endocrine status, and APOE genotype impact inflammatory processes across AD progression from risk to late stage disease.

## Age-Related Neuroinflammation

Aging has a broad systems-level effect on human biology, which is evidenced by alterations in physiological function, metabolism, cognition, and inflammation (Smith et al., 2005). The effects of aging on immune responses are extensive and complex. In some individuals, adaptive immune responses will decline with age, whereas, others will experience aberrant immune responses leading to autoimmune disorders (Goronzy and Weyand, 2012; Vadasz et al., 2013; Fougère et al., 2017). Aging is associated with accumulation of oxidative stress and DNA damage and chronic low-grade inflammation (Cui et al., 2012). Though the effect of aging on cognitive function is variable, age remains the greatest risk factor for Alzheimer’s in which inflammation is an early and persistent hallmark of the disease (von Bernhardi et al., 2015).

Central nervous system microglia plays a prominent role in innate immunity. Microglia constantly conducts surveillance of brain parenchyma to detect foreign pathogens and clear debris (Streit et al., 2004; von Bernhardi et al., 2015). Microglia detects and responds to a broad range of triggers including TBI, infections and damage associated molecular patterns (DAMPs). Reactive oxygen species (ROS), extracellular DNA and ATP all act as DAMPs (von Bernhardi et al., 2015; Gulke et al., 2018).

**FIGURE 1:**
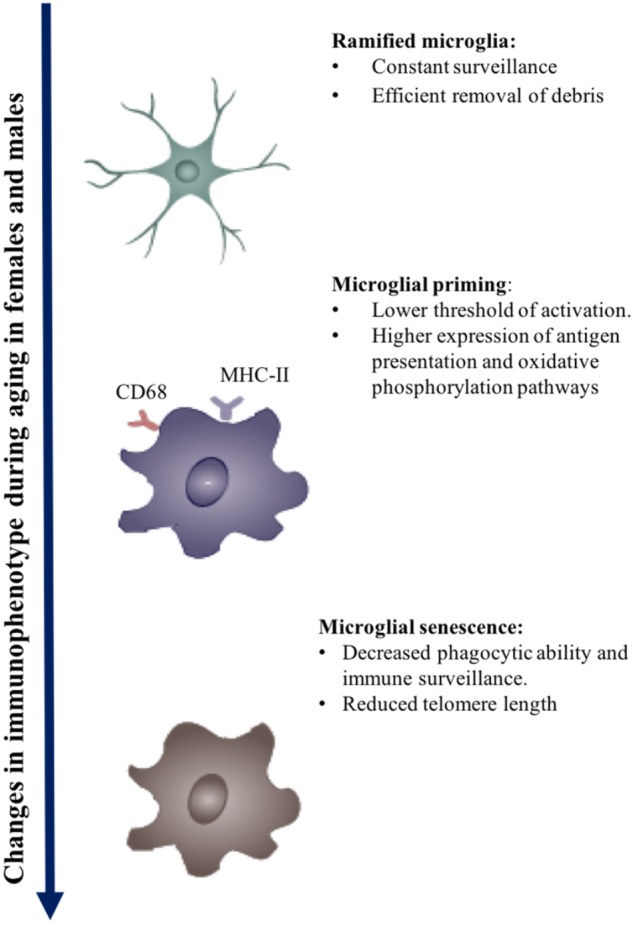
Effect of aging on microglial immunophenotype. Changes in the immunophenotype of aging. Early to midlife aging can induced microglial priming, which is evident in the upregulation of MHC-II molecules, Fcg receptors, and microglial receptors. Chronic activation may eventually lead to immune senescence.

**FIGURE 2:**
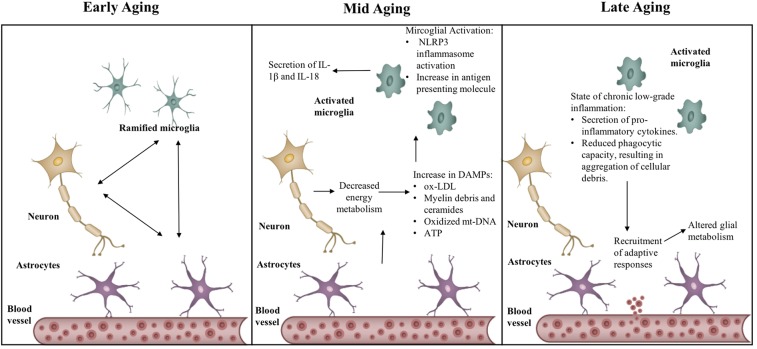
Transitions in the inflammatory phenotype across aging. Aging causes activation of neuro-inflammatory mechanisms – NLRP3 inflammasome and increased MHC-II expression, through production of sterile triggers, such as, oxidized low-density lipoprotein (Ox-LDL), myelin debris, oxidized mitochondrial DNA (mt-DNA), ATP. State of chronic low-grade inflammation leads to recruitment of adaptive responses, in turn affecting glial metabolism.

Innate immune responses by microglia are phenotypically typified by enlargement of the cell body, and molecularly by the upregulation of CD68, major histocompatibility complex-II (MHC-II) along with costimulatory molecules and secretion of pro and anti-inflammatory cytokines (Kim and Joh, 2006). The onset of innate immune responses leads to activation of the adaptive immune response. The innate activation of the adaptive immune response results in infiltration of peripheral immune cells, particularly T cell invasion of the brain (Kim and Joh, 2006). Together the innate and adaptive immune responses create the chronic low-grade inflammation typical of aging (Kim and Joh, 2006; see **Figures 1**, **2**).

Microglial phenotype is dynamic. It changes with age and is typified by both homeostatic and disease phenotypes (Butovsky et al., 2014; Keren-Shaul et al., 2017; Krasemann et al., 2017). The telomerase deficient accelerated aging mouse model exhibits reduced microglial numbers and deficient morphological and cellular processes (Khan et al., 2015). Further, microglial response to activation is stage of development dependent. Production of cytokines and trophic factors by microglia increases linearly with age (Lai et al., 2013). Microglia isolated from younger mice (2–4 months) exhibit a lower expression of proinflammatory cytokines: TNFα, IL-6, and IL-1β than older mice (Helenius et al., 1996; Crain et al., 2013; Latta et al., 2015). On activation by ATP, microglia isolated from neonatal rats and 13–15 month old adult rats have a more robust inflammatory response exhibited by upregulation of nitric oxide, TNFα, and brain derived neurotrophic factor (BDNF) in comparison to microglia derived from younger animals (2–11 months) (Lai et al., 2013). Aging distinctly affects migratory function of microglia; younger microglia on encountering activating signals exhibit an increase in motility and rapid extension of ramifications, whereas older microglia are less dynamic. Transcriptomic studies corroborate a reduction in migratory ability of microglia with age as age affects actin cytoskeleton reogranization which is vital in both phagocytosis and migration (Damani et al., 2011; Orre et al., 2014).

Comparison of the transcriptomic profiles of young and aged microglia revealed that microglial receptors (Trem2c, P2yr12, P2yr13, and Adora) involved in recognizing DAMPs such as, oxidized low-density lipoprotein, mitochondrial DNA, extracellular ATP decreased with age (Orre et al., 2014). In contrast, the expression of receptors that recognize pathogens and microbes (Tlr2, CD74, Ltf, Clec7a, Cxcl16, and Ifitm6) increases with age (Orre et al., 2014). Age-related changes in microglial transcriptome are not ubiquitous as the expression of phagocytic receptors (Cd14, Cd68, Cd11b, and ICAM) remained unaltered in aging (Hickman et al., 2013; Smith and Dragunow, 2014). However, activation of microglial phagocytosis is diminished in aging. Studies characterizing microglial phagocytic capacity, report a reduction in phagocytosis with age which is especially evident following activation (Li M.D. et al., 2015; Ritzel et al., 2015). These findings indicate that despite stable expression of phagocytic receptors, the functional capacity of microglia decreases with age (Li M.D. et al., 2015; Ritzel et al., 2015). For example, the ability of microglia to phagocytose Aβ is affected by age, with microglia isolated from postnatal animals effectively phagocytosing Aβ fibrils, whereas adult microglia lose their capacity to do so (Floden and Combs, 2011). Other contributing factors to microglial senescence are age-related myelin degeneration and lysosomal storage in microglia, which in turn burden microglial clearance function (Holtman et al., 2015; Safaiyan et al., 2016).

Systemic inflammation and aging cause microglial priming. Primed microglia have a lower threshold for activation, are hypersensitive, develop an exaggerated immune response on activation, and have a distinct molecular signature from the M1–M2 phenotype (Perry and Teeling, 2013; Holtman et al., 2015; Ojo et al., 2015). The molecular signatures of primed microglia include the overexpression of antigen presentation, redox pathways, oxidative phosphorylation, and lysosomes.

In summary, during aging primed microglia generate a pro-inflammatory cascade due to their lower threshold of activation, enhanced reactivity, and limited functional capacity on encountering secondary triggers. The chronic activation of microglia coupled with age-related microglial priming hastens the process of senescence to cause loss of function over time (Franceschi et al., 2000; Streit and Xue, 2014). Senescent microglia appear to have lesser ramifications and stouter cell bodies, often referred to as dystrophic microglia.

### Immunometabolic Sensor of Aging: Targeting Aging as a Disease

The inflammasome complex is a sensor of DAMPs. DAMPs act as an inflammatory challenge to the host defense mechanism and lead to the activation of the NLRP3 (Nod-like receptor pyrin domain 3) inflammasome complex (Youm et al., 2013; Zhang et al., 2013). Within the family of innate inflammasome sensors, the NLRP3 inflammasome has the unique ability to detect sterile inflammatory triggers. It can detect a wide range of metabolic and aging-related DAMPS, such as ROS production, glucose tolerance, and insulin resistance (Vandanmagsar et al., 2011; Salminen et al., 2012; Walsh et al., 2014), lipotoxic fatty acids, ceramides, free cholesterol, uric acid, and ATP, and it releases IL-1β and IL-18 (Youm et al., 2012, 2013; Zhang et al., 2013; see **Figure 2**).

The NLRP3 inflammasome complex activation is a two-step process. Molecular pathogens like lipopolysaccharides (LPS) have been shown to prime cells, leading to the activation of pattern recognition receptors (PRRs), the release of IL-1β, and increased expression of NLRP3. When followed by a secondary trigger such as ATP, this process causes the inflammasome to assemble and causes further activation. NLRP3 is also activated by the accumulation of damaged mitochondria due to the inhibition of autophagy, resulting in excessive production of ROS. oxidized mitochondrial DNA is also implicated in the activation of NLRP3 (Dixit, 2013). The activation of NLRP3 by ROS is mediated by thioredoxin interacting protein (TXNIIP) (Youm et al., 2012, 2013; Zhang et al., 2013).

The activation patterns of NLRP3 are similar in both macrophages and microglia. NLRP3 activation leads to the priming of microglia and reducing the threshold for activation (Halle et al., 2008; Heneka et al., 2013). Increase in caspase-1 activity in postmortem MCI and AD brains indicates the possible participation of the NLRP3 inflammasome in AD pathogenesis (Heneka et al., 2013). Targeting NLRP3 and NF-κB (nuclear factor kappa-light-chain-enhancer of activated B cells) has been associated with a reduction in pathology of AD (Tang et al., 2015). In animal models carrying AD pathology, NLRP3 knockout and caspase-1 knockout caused spatial memory to remain intact. Moreover, the microglial phenotype in the NLRP3 knockout model shifted to the M2 anti-inflammatory phenotype with greater neuroprotection improved clearance of the plaque burden (Halle et al., 2008; Heneka et al., 2013).

With several studies linking NLRP3 inflammasome activation to chronic low-grade inflammation observed in aging, therapeutics targeting this sensor has also emerged. In a recent study, the ketone body – β-hydroxybutyrate (BHB) was found to suppress NLRP3 activation caused by urate crystals, lipotoxic fatty acids, and ATP. The levels of BHB increase with starvation, caloric restriction, and high-intensity exercise. Aging also marks a shift in the fuel usage and dependence on different fuel mechanisms. The inhibition of NLRP3 by BHB resulted in a decrease of IL-1β and IL-18 production by monocytes. BHB also reduced caspase-1 activation and IL-1β secretion in mouse models of NLRP3-mediated chronic inflammatory diseases like Muckle-Wells syndrome (Youm et al., 2015).

Another molecule, MCC950, blocked canonical and non-canonical activation of NLRP3 and attenuated experimental autoimmune encephalomyelitis (EAE). Both, MCC950 and BHB, were used in a mouse model of Muckle-Wells syndrome, which is characterized by chronic inflammation mediated by NLRP3. Thus, targeting NLRP3 in aging and aging-related disorders could be an important therapeutic strategy (Coll et al., 2015).

## Menopause, Inflammation, and AD

The endocrine transition of the perimenopause to the post-menopause, while associated with loss of reproductive function (Brinton et al., 2015), is also associated with rise in chronic low-grade inflammation (Yin et al., 2015). Chronic systemic inflammation accelerates ovarian failure (Ağaçayak et al., 2016). Conversely, depleting proinflammatory cytokines IL-1α and IL-1β extends ovarian function and lifespan (Uri-Belapolsky et al., 2014). Concurrent with the chronic low-grade inflammation, the perimenopausal transition is typified by decline in brain glucose metabolism and mitochondrial respiration (Yao et al., 2010; Ding et al., 2013; Brinton et al., 2015; Yin et al., 2015; Mosconi et al., 2017a,b), myelin catabolism (Klosinski et al., 2015) and loss of white matter volume (Mosconi et al., 2017a,b), beta-amyloid deposition in brain (Mosconi et al., 2017a,b) and changes in neurological function (Brinton et al., 2015). Later age at natural and surgical menopause is associated with better verbal memory (Kuh et al., 2018). Surgically induced menopause prior to natural menopause is associated with rapid cognitive decline and earlier onset of AD (Rocca et al., 2008; Bove et al., 2014). Further, studies have shown that post-menopausal women with higher estradiol levels have a reduced risk of developing AD (Manly et al., 2000).

Post-menopausal women are at higher risk for developing autoimmune disorders and obesity (Doran et al., 2002; Bove, 2013). The incidence of RA is higher in peri- and post-menopausal women (Doran et al., 2002). The pathology of multiple sclerosis worsens after menopause (Tutuncu et al., 2013). Post-menopausal women are more prone to robust immune responses. The lack of ovarian steroidal hormones potentiates the inflammatory process predisposing menopausal women to immune disorders (Benedusi et al., 2012; Kireev et al., 2014; Sharma et al., 2018). Menopause and the associated lack of steroidal hormones further potentiate inflammation, which is reflected in levels of circulating cytokine levels and inflammatory responses. IL-6 and sIL-6 levels are higher in postmenopausal women (Giuliani et al., 2001). IL-4 and IL-2 levels also increase with menopause, which can be reversed by hormone therapy (Yasui et al., 2007). Serum IFN-γ levels increase during early menopause but decrease in later menopause (Goetzl et al., 2010). Peripheral blood mononuclear cells (PBMCs) isolated from postmenopausal women produced higher IL-6, IL-1β, and TNFα upon induction by LPS than PBMCs isolated from premenopausal women (Brooks-Asplund et al., 2002).

In addition to an altered cytokine profile, changes in T cell biology occur in women during this endocrine transition. Pre-menopausal women have higher CD4 counts than men and thus a more robust response to vaccination. Menopause causes a reduction in CD4 T cell numbers. This eventually causes an inversion of the CD4/CD8 T cell ratio, which is indicative of aging and can be correlated with increased oxidative stress (Larbi et al., 2008; Gameiro et al., 2010; Muller et al., 2015). The number of B2 cells (involved with antibody production) also decreases with menopause, especially during late menopause in comparison to perimenopause (Kamada et al., 2001). In mice, ovariectomy causes a reduction in the LPS-induced proliferation of leukocytes and subsequent chemotaxis, which is indicative of premature immune senescence (Baeza et al., 2011). Ovariectomized animals generally have a reduced and delayed adaptive response to vaccination, leading to decreased IgG titers in comparison to animals with intact ovaries (Haberthur et al., 2010). These changes are indicative of immune senescence occurring during menopausal transition. In the context of AD, the systemic effect of menopause on inflammation combined with effects on neurological function indicates cruciality of the menopausal transition in AD pathogensis.

### Molecular Neuro-Inflammatory Mechanisms in Menopause

Menopause is composed of three transitions; the perimenopause that precedes menopause, the cessation of reproductive capacity, menopause, and the years following menopause, post-menopause. Concomitant with this endocrine aging is chronological aging as the endocrine transition states span multiple years. Each of these endocrine stages is characterized by complex hormonal fluctuations (Brinton et al., 2015).

Decline in estradiol level during perimenopause and menopause coincides with a bioenergetic deficit in brain (Ding et al., 2013). Estradiol is a master regulator of metabolic function in the female (Rettberg et al., 2014). Clinical evidence of decline in glucose metabolism in brain and the coincident bioenergetic deficit is evidenced by reduced uptake of 18-fludeoxyglucose detected by positron emission tomography (PET) in perimenopausal and menopausal women (Mosconi et al., 2017a,b). The bioenergetic deficit precedes a shift to utilization of ketone bodies as a compensatory response to decline in brain glucose as bioenergetic fuel to generate ATP in brain. This shift to utilizing an auxiliary fuel during female endocrine aging activates catabolism of white matter as an endogenous lipid source of ketone bodies in brain (Klosinski et al., 2015) and a concomitant increase in microglial and astrocytic reactivity (Xie et al., 2013; Suenaga et al., 2015).

**FIGURE 3:**
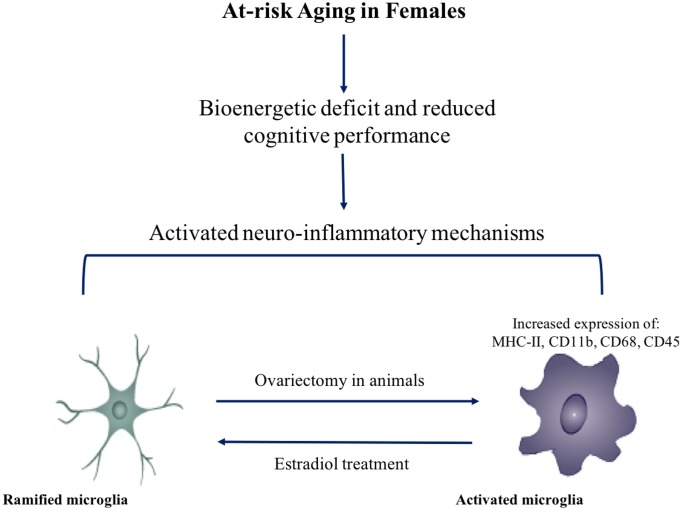
Effect of menopause on immunophenotype. Decline in ovarian hormones induces a pro-inflammatory state in microglia. Activation of microglia and neuro-inflammatory mechanisms may be involved in at-risk aging females.

Analyses of microarray data obtained from the brains of postmenopausal women made available by NCBI revealed an inflammatory gene expression profile in the post central and superior frontal gyrus (Sárvári et al., 2012). In comparison to pre-menopausal women, post-menopausal women showed an upregulation in microglial markers CD14, CD18, and CD45, as well as TLR4 and MHC-II markers CD74 and C3 (Sárvári et al., 2012). These findings in the human female brain were consistent with the pattern of gene expression in the frontal cortex of ovariectomized middle-aged rats (13 months old) (Sárvári et al., 2012). Ovariectomy caused an upregulation of microglial reactivity markers CD11b, C18, CD45, and CD86, complement pathway C3, and phagocytic markers Msr2 and CD32. Together, these data are indicative of a shift in the microglial phenotype to an activated state (see **Figure 3**).

Hippocampal inflammatory gene expression in middle-aged rats drastically changed upon ovariectomy. A similar upregulation of microglial markers (CD45, IBA1, CD68, CD11b, CD18, Fcgr1a, and Fcgr2b) to that witnessed in the cortex was observed in the hippocampus. The gene expression results imply a possible activation of microglia. This effect was mitigated by treatment with estradiol and selective estrogen receptor-α (ERα) and estrogen receptor-β (ERβ) agonists (Sarvari et al., 2015). Human post-menopausal gene expression in the hippocampus corroborated the inflammatory gene expression pattern in ovariectomized rats, with an upregulation of microglial reactivity markers CD11b, CD18, IBA1, CD14, and complement C3 (Sarvari et al., 2015).

In parallel, aging is associated with a marked upregulation in genes encoding the major histocompatibility complex class I and class II (MHC-I and MHC-II) (VanGuilder et al., 2011), alterations in Toll-like receptors (TLRs) (Shaw et al., 2011), and the complement pathway (Reichwald et al., 2009). This effect is more pronounced in women and represents the sexual dimorphism of the immune system (Blalock et al., 2003; Berchtold et al., 2008). The dynamics involved between age and menopause-related increase in myelin degeneration and microglial priming can be a tipping point in the neuro-inflammatory system. The increased myelin antigen load and upregulation of antigen presentation by microglia can set forth a cascade that leads to dysregulated glial metabolism and hypertrophy, eventually causing altered extracellular matrix (Blalock et al., 2003). Each event is pivotal in the development of AD.

### Hormone Therapy: A Potential Anti-inflammatory Preventive Intervention for Alzheimer’s Disease?

Hormone therapy (HT) promotes neuronal survival and has been shown to improve cognitive function and episodic memory in perimenopausal and postmenopausal women (Morrison et al., 2006; Brinton, 2008). Epidemiological studies have shown that HT delays the onset of AD as well as reduces the risk of developing AD (Tang et al., 1996; Persad et al., 2009; Dye et al., 2012). Women transitioning through their menopause benefit most from HT as compared to women who have already transitioned (Girard et al., 2017). Results from several clinical trials have emphasized on the timing of treatment with HT and the drawbacks of missing the window of treatment (Hogervorst et al., 2000; Zandi et al., 2002; Shumaker et al., 2003). This effect of estradiol in HT has been explained by two theories: the healthy cell bias of estrogen action (Brinton, 2008) and critical window hypothesis (Maki, 2013). Healthy cell bias highlights that neuronal viability and health at baseline are important for estradiol to exert its therapeutic efficacy, whereas the critical window hypothesis focuses on the perimenopausal transition, when cells are still healthy, being a key phase for using HT. The use of estradiol in HT provides a therapeutic opportunity to target inflammatory pathways that simultaneously modulate metabolic functions, thereby providing a supportive milieu for neuronal survival and growth (Vegeto et al., 2008; Zhao et al., 2014). HT restores the hormonal levels in post-menopausal women to those of premenopausal women. Post-menopausal women using HT have higher lymphocyte numbers and higher circulating monocytes in comparison to post-menopausal women who are not on HT (Kamada et al., 2000). Likewise, levels of B2 cells involved in antibody production are significantly higher in HT users in comparison to non-users (Kamada et al., 2001).

Estrogen receptor alpha (ERα) and estrogen receptor beta (ERβ) are abundantly expressed in astrocytes, microglia, and neurons, and both ERα and ERβ are involved in regulating the immunomodulatory responses exerted by astrocytes and microglia (Liu et al., 2003; Khan and Ansar Ahmed, 2015). In ovariectomized middle-aged rats, estradiol induces downregulation of the complement pathway and macrophage-associated genes in the frontal cortex. This effect was mediated through ERα and ERβ (Sárvári et al., 2011). Estradiol treatment in microglial cells induces a dose-dependent attenuation in superoxide release, phagocytic activity, and a concomitant increase in iNOS activity, without altering NF-κB expression (Bruce-Keller et al., 2000; Drew and Chavis, 2000). Some studies have shown that sex steroids reduce neuroinflammation via inhibiting the inflammasome complex, a possible downstream effect mediated by ERα and ERβ (Slowik and Beyer, 2015).

Astrocytes also participate in mediating the neuroprotective anti-inflammatory effect of estradiol via ERα (Spence et al., 2011). In contrast to microglial cells, estradiol inhibits NF-κB expression in astrocytes (Giraud et al., 2010; Acaz-Fonseca et al., 2014). Estradiol inhibits secretion of proinflammatory cytokines IL-6, TNF-α, IL-1β, expression of matrix metalloproteinases 9 (MMP-9), and interferon gamma-inducible protein 10 (IP-10) in astrocytes. Estradiol also reduces proinflammatory cytokines secreted by astrocytes when exposed to Aβ (Giraud et al., 2010; Acaz-Fonseca et al., 2014).

Much like estradiol, selective estrogen receptor modulators (SERMs) exert a neuroprotective effect by reducing neuroinflammation. Tamoxifen and raloxifene both reduce microgliosis, astrogliosis, and the production of proinflammatory cytokines IL-6 and IP-10 induced by LPS administration (Arevalo et al., 2012). They have also been demonstrated to protect neurons against neurotoxicity caused by neuroinflammation through an ER mediated pathway (Ishihara et al., 2015). SERMs reduce the proinflammatory response produced by astrocytes and are helpful in potentiating their neurotrophic function (Tapia-Gonzalez et al., 2008; Cerciat et al., 2010; Arevalo et al., 2012; Ishihara et al., 2015).

## Apoeε4, Inflammation, and Alzheimer’s Disease

APOEε4 is the primary genetic risk factor for the late onset form of AD (Scacchi et al., 1995; Liu et al., 2014; Manning et al., 2014). The human form of the APOE gene possesses three polymorphic alleles: E2, E3, and E4. The E3 allele occurs more frequently (77%) than E2 (8%) and E4 (14%) (Eisenberg et al., 2010). The E4 allele occurs in 40% of AD patients (Farrer et al., 1997). However, 91% of homozygous E4 carriers and 47% heterozygous carriers go on to develop AD (Corder et al., 1993).

Female APOEε4 carriers are more susceptible to developing AD than males (Altmann et al., 2014; Ungar et al., 2014; Neu et al., 2017). Prospective cohort studies have also suggested that female APOEε4 carriers are at greater risk of converting from MCI to AD than males (Altmann et al., 2014). Female APOEε4 carriers also have a higher rate of cognitive decline than APOEε3 carriers (Holland et al., 2013). Leukocyte telomere length is also greatly reduced in APOEε4 carrier’s relative to age-matched controls, reflecting premature aging of female APOEε4 carriers (Jacobs et al., 2013).

Apolipoprotein E (ApoE) is a key regulator of lipid homeostasis and in the brain, it functions to shuttle lipid molecules from astrocytes and microglia to neurons, via lipoprotein complexes (Liu et al., 2013). In the periphery, ApoE is expressed in macrophages and liver. In the central nervous system, ApoE is mainly produced by astrocytes and microglia (Liu et al., 2013).

### Sources of Neuroinflammation in APOEε4

ApoE is known to exert an immunosuppressive effect by inhibiting lymphocyte proliferation, Ig synthesis, and neutrophil activation. ApoE also exerts this immunosuppressive property on microglial activation (Guo et al., 2004; Baitsch et al., 2011; Christensen et al., 2011). Relative to APOEε2 and APOEε3 carriers, APOEε4 carriers generate less ApoE protein in periphery and brain (Larson et al., 2000). Given the reduced amounts of ApoE protein in APOEε4 carriers, this population could be predisposed to a heightened inflammatory response when encountering a sterile inducer or infection (Ukkola et al., 2009; Gale et al., 2014; Tai et al., 2015).

Due to the differences in cysteine and arginine residues translated at positions 112 and 158, each of the three different isoforms of APOE exhibit different conformations. The conformational change in the protein affects its stability, folding characteristics, and the propensity to bind lipoprotein particles (Jofre-Monseny et al., 2008). APOEε4 has a globule-like structure and preferably binds to very low-density lipoprotein (vLDL) and low-density lipoprotein (LDL) particles. However, APOEε2 and APOEε3 tend to bind high-density lipoprotein (HDL) particles (Jofre-Monseny et al., 2008). This difference in protein structure affects the lipid shuttling ability of ApoE, which leads to hypercholesterolemia in APOEε4 carriers and increases the predisposition for generation of plaques (Jofre-Monseny et al., 2008). ApoE also exerts an inhibitory effect on the oxidation of LDL in an isoform-specific manner (E2>E3>E4) (Miyata and Smith, 1996). Among smokers, APOEε4 carriers have significantly higher amounts of oxidized LDL (ox-LDL) (Jofre-Monseny et al., 2008).

Cholesterol, ox-LDL, and Aβ are sterile inducers of inflammation called DAMPs (Miller et al., 2011; Clark and Vissel, 2015). DAMPs are recognized by PRRs expressed on macrophages, dendritic cells, monocytes, microglia, and neutrophils, which trigger the activation of an inflammatory process (Silverstein and Febbraio, 2009). One such PRR is CD36. In the context of recognizing DAMPs and plaque formation, it was recently demonstrated that CD36 (expressed on monocytes, macrophages, and microglia) recognizes soluble ligands such as oxidized LDL and soluble Aβ and converts them to crystals and fibrils, respectively. This leads to the assembly and activation of the NLRP3 inflammasome and the consequent release of the proinflammatory cytokine IL-1β (Sheedy et al., 2013; Oury, 2014).

Due to the increased probability of APOEε4 carriers to develop plaques, cholesterol crystals, and amyloid depositions, cellular immune function and reactivity are affected. In APOEε4 carriers there is a reduced clearance and efflux of cholesterol from macrophages (Cash et al., 2012). Moreover, increased nitric oxide production in APOEε4 causes increased platelet aggregation and secretion of adhesion molecules, further enabling the plaque formation in the periphery. In the brain, microglial and astrocytic clearance of debris is also diminished (Guo et al., 2006).

ApoE also affects Aβ uptake and oligomerization and thus can be a key factor in Aβ turnover. AD patients possessing the APOEε4 allele were found to have higher levels oligomeric Aβ in their brains as compared to APOEε3 carriers, implicating an association between ApoE with Aβ (Hashimoto et al., 2012). *In vitro* experiments correspond to clinical findings and have shown that APOEε4 has the greatest effect on promoting Aβ oligomerization in comparison to other isoforms (Hashimoto et al., 2012). Blocking Aβ and ApoE interaction by ApoE Aβ antagonist in hippocampal neuronal and astrocytic co-culture systems led to decreased accumulation and oligomerization of Aβ. Treatment with ApoE Aβ antagonists also inhibited the loss of synaptic proteins induced by Aβ accumulation (Kuszczyk et al., 2013; Liu S. et al., 2014).

Coupled with the Aβ oligomerization, ApoE also affects Aβ uptake. Astrocytes secrete ApoE as a lipoparticle into the interstitial fluid, where it binds with Aβ. Neurons endocytose and internalize these lipoparticles, thus promoting Aβ uptake. APOEε4 isoform has maximal binding affinity to Aβ and thus causes a greater uptake of Aβ by neurons in comparison to other isoforms (Mulder et al., 2014). APOEε4 prevents the uptake of oligomeric Aβ by astrocytes and the uptake of both oligomeric and fibrillar Aβ by microglia thereby inhibiting its clearance (Mulder et al., 2014).

ApoE also modulates Aβ clearance by microglia by regulating Aβ clearing enzymes such as neprilysin intracellularly and insulin degrading enzyme extracellularly. Effective degradation of Aβ depends on Liver X Receptor (LXR) activation, the isoform of APOE expressed, and the lipidation status of ApoE particles (Hashimoto et al., 2012; Kuszczyk et al., 2013; Liu S. et al., 2014). Activation of LXR/RXR (Retinoid X receptor) potentiates Aβ clearance as it compensates for the loss of APOEε4 function and induces the expression of ATP-binding cassette transporter subfamily A member 1 (ABCA1) and ApoE, thus inducing clearance by microglia and astrocytes (Lefterov et al., 2007; Terwel et al., 2011; Lee et al., 2012; Mandrekar-Colucci et al., 2012; Tai et al., 2014). Therefore, the presence of APOEε4 promotes the production of Aβ and uptake by neurons while preventing clearance and enabling the production of DAMPs and chronic low-grade inflammation.

**FIGURE 4:**
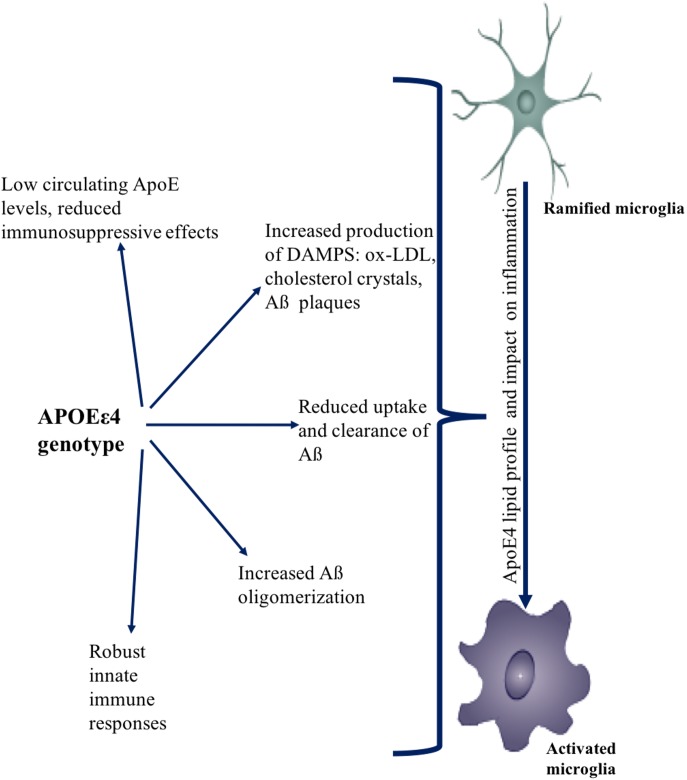
Effect of APOEε4 on immune function. APOEε4 genotype affects the uptake, clearance, and production of sterile triggers of inflammation. APOEε4 causes increased accumulation of DAMPs such as Aβ, ox-LDL, cholesterol. APOEε4 also causes more robust innate immune responses. Collectively, the effects of APOEε4 genotype promotes an overall neuro-inflammatory state.

Given the dysregulated lipid metabolism and impairment of cellular function with age, the APOEε4 allele is associated with increased systemic inflammation. It is expected that this would be reflected in cytokine levels, which are inflammatory markers such as C-Reactive Protein (CRP) measured in plasma/serum. Surprisingly, however, this is not the case, at least with CRP. Studies have consistently shown that CRP levels are lower in APOEε4 carriers (Ukkola et al., 2009; Lima et al., 2014; Metti et al., 2014; Yun et al., 2015). Marz et al. (2004) proposed that the metabolism of CRP might be associated with the mevalonate/cholesterol synthetic pathway, which might be downregulated in APOEε4 carriers. On the other hand, studies have shown that levels of IL-1β and vascular inflammatory marker: vascular cell adhesion molecule-1 (VCAM-1) are higher in E4 carriers (Olgiati et al., 2010; see **Figure 4**).

The APOEε4 allele also accelerates aging, which is reflected in the shorter telomeres in APOEε4 women in comparison to APOEε3 women (Jacobs et al., 2013). The accelerated aging phenotype is also evident in the reduction of T cell numbers. Age-related reduction in T cells for women is more dramatic during menopause, which is even more pronounced if the woman is an APOEε4 carrier (Begum et al., 2014). In comparison to APOEε3 derived microglia, estradiol has a reduced anti-inflammatory effect on microglia derived from APOEε4 (Brown et al., 2008). The APOEε4 allele is also a risk factor for metabolic syndrome and has been associated with RA, thus increasing the risk of comorbidities that can, in turn, affect systemic inflammation (Sima et al., 2007; Gungor et al., 2012; Toms et al., 2012; Johnson et al., 2013). APOEε4 also affects immune function by causing blood barrier dysfunction (Nelson et al., 2016).

### Innate Immune Responses in APOEε4

Inflammatory responses triggered by innate immune agonists are highest in APOEε4 carriers (Vitek et al., 2009; Gale et al., 2014). This finding holds true in cells isolated from both humans and rodents and in both the periphery and the brain (Gale et al., 2014; Li X. et al., 2015). Therefore, inflammatory triggers such as TBI, infection, and DAMPs produced from metabolic syndrome significantly increase the inflammatory response in APOEε4 carriers. This may potentially lead to the incomplete resolution of inflammation to initiate chronic inflammatory processes that result in neurotoxicity. This trend is evident in HIV-associated dementia, for which E4 carriers have increased risk (Chang et al., 2011).

A heightened inflammatory response occurred in microglia derived from humanized APOEε4 knock-in mice upon on treatment with the TLR3 and TLR4 activator LPS compared to APOEε3 mice (Vitek et al., 2009; Heneka et al., 2015). The inflammatory response was characterized by altered cell morphology, increased nitric oxide production, COX-2 expression, prostaglandin E2 (PGE2) expression, and cytokine production (IL-6, TNF-α, and IL-12p40). In contrast, TREM2 expression was decreased (Vitek et al., 2009; Heneka et al., 2015). A comparable inflammatory response was observed in peripheral macrophages isolated from APOEε4 mice. The E4 allele increases the reactivity of glial and peripheral immune cells, thus aggravating the neurotoxic proinflammatory response (Vitek et al., 2009; Heneka et al., 2015).

## Discussion

**FIGURE 5:**
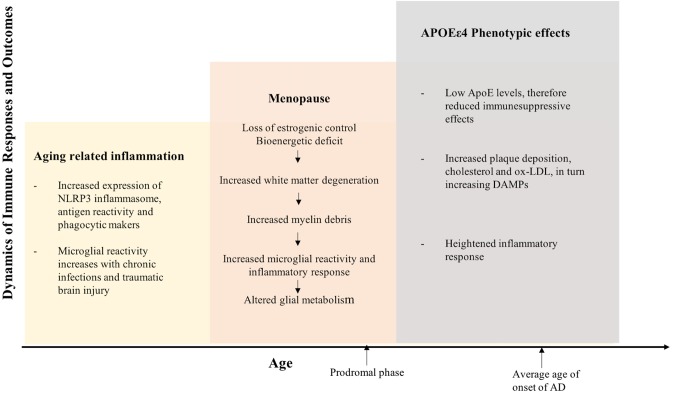
Inflammation integrates Alzheimer’s disease risk factors of female sex, chronological age, endocrine aging, and APOEε4 genotype. The three hit model of Alzheimer’s risk: aging, menopause, and APOEε4 genotype collectively induce a compromised bioenergetic system in brain that is impacted by the chronic low grade innate inflammation of aging coupled with APOEε4 dysregulated cholesterol homeostasis lead to activation of the adaptive immune response. The inflammatory immune response is the factor that bridges across each of the risk factors for AD. Immune system regulators that are specific to stage of disease and inflammatory phenotype would provide a therapeutic strategy to disconnect the bridge that drives disease.

**FIGURE 6:**
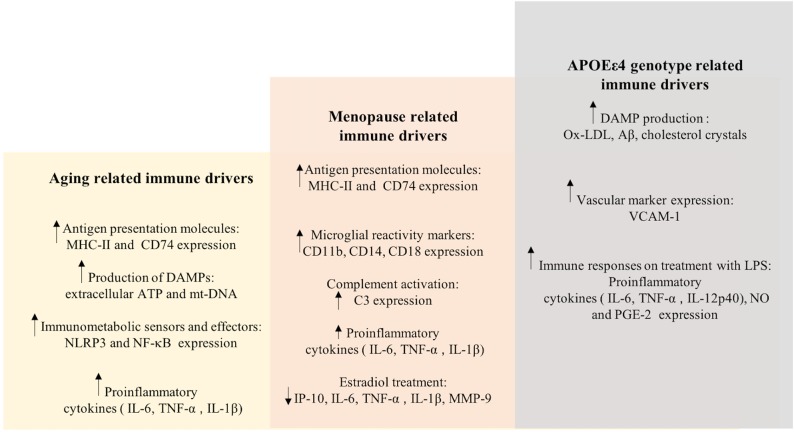
Immune drivers involved in aging, menopause, and APOEε4 genotype related inflammation. Key immune drivers contributing to inflammation due to aging, menopause and APOEε4 genotype related inflammation are detailed to give a global picture of immune dynamics (upward and downward arrows indicate increased and decreased expression, respectively).

The etiology of the prodromal phase of AD presents as a complex interplay between several risk factors, which is relevant to therapeutic interventions and preventive strategies (see **Figures 5**, **6**). This implies that therapeutic strategies should employ stratification of patient populations regarding parameters of age, sex, and APOE genotype. It also calls for the use of combination therapies that modulate inflammation, lipid-based metabolism in APOEε4 carriers, and loss of estrogenic control in menopausal women. For example, preventive strategies to reduce age-related inflammation could include a combination of NSAIDs and statins in middle-aged adults (45–55 years). In women, this therapy could be modified to include HT during their perimenopausal transition. Patient’s medical histories and electronic health records are a source of indicators for chronic inflammation. These strategies should be tailored to the patient’s metabolic profile, genetic history, and endocrine-related transition states (see **Figures 5**, **6**).

This understanding of the disease progression also calls for change in design of clinical trials that target the amyloidogenic pathway and treat later stages of the disease pathogenesis. Trial design should incorporate the identification of persons with an increased risk of developing AD, and utilize a risk-factor-based responder analysis. Inflammation-mediated therapeutic and preventive strategies will largely depend upon this stratification of patient populations.

The inflammatory response is influenced by age, chromosomal sex, endocrine transition – menopause and APOE genotype. Inflammation is characteristic of each of these modifying factors and can be a driving force for development of AD. Thus, inflammation has been a therapeutic target in multiple clinical trials for AD. However, each of these trials have failed to meet primary endpoints. Reviewed herein is a consideration of the multiple factors that contribute to and modify the inflammatory phenotype. Going forward, in both discovery and clinical science, it will be important to delineate the etiology of the inflammatory response, the stage of the inflammatory cascade, and the activated network of inflammatory signaling. Inflammation is a moving target and thus requires a precision approach to identifying etiology, stage and appropriate therapeutic target.

## Concluding Remarks

In summary, neuro-inflammatory processes are evident early in the latent prodromal phase and worsen during the course of the Alzheimer’s. Disparate results from epidemiological and clinical trials targeting inflammation, highlight the complexity of the inflammatory process. The inflammatory processes that occur during aging, midlife endocrine transitions, and in the APOEε4 carrier contribute to risk and progression of AD. The chronic inflammatory processes that are activated during midlife chronological and endocrine aging, ultimately limit the clearance capacity of microglia and lead to immune senescence. The loss of estrogenic control of bioenergetic function in the brain coupled with dysregulated lipid metabolism in the APOEε4 genotype adversely impact microglial function and clearance mechanisms. The dynamic and context specific activation pattern of the inflammatory processes provide plausible mechanisms underlying failed clinical trials of anti-inflammatory agents in Alzheimer’s patients. Collectively, these considerations highlight the rationale for stratifying AD clinical trial cohorts based on their inflammatory phenotype. Combination therapies that include targeted use of anti-inflammatory agent’s specific to the immune phenotype could have a higher probability of successfully modifying risk and progression of Alzheimer’s disease.

## Author Contributions

AM and RB wrote and reviewed the manuscript.

## Conflict of Interest Statement

The authors declare that the research was conducted in the absence of any commercial or financial relationships that could be construed as a potential conflict of interest. The reviewer AG and handling Editor declared their shared affiliation at the time of the review.
